# A Linked Open Data–Based Terminology to Describe Libre/Free and Open-source Software: Incremental Development Study

**DOI:** 10.2196/38861

**Published:** 2023-01-20

**Authors:** Franziska Jahn, Elske Ammenwerth, Verena Dornauer, Konrad Höffner, Michelle Bindel, Thomas Karopka, Alfred Winter

**Affiliations:** 1 Institute of Medical Informatics, Statistics and Epidemiology Faculty of Medicine Leipzig University Leipzig Germany; 2 Institute of Medical Informatics University for Health Sciences, Medical Informatics and Technology Hall in Tirol Austria; 3 GNU Solidario Las Palmas de Gran Canaria Spain

**Keywords:** health informatics, ontology, free/libre open-source software, software applications, health IT, terminology

## Abstract

**Background:**

There is a variety of libre/free and open-source software (LIFOSS) products for medicine and health care. To support health care and IT professionals select an appropriate software product for given tasks, several comparison studies and web platforms, such as Medfloss.org, are available. However, due to the lack of a uniform terminology for health informatics, ambiguous or imprecise terms are used to describe the functionalities of LIFOSS. This makes comparisons of LIFOSS difficult and may lead to inappropriate software selection decisions. Using Linked Open Data (LOD) promises to address these challenges.

**Objective:**

We describe LIFOSS systematically with the help of the underlying Health Information Technology Ontology (HITO). We publish HITO and HITO-based software product descriptions using LOD to obtain the following benefits: (1) linking and reusing existing terminologies and (2) using Semantic Web tools for viewing and querying the LIFOSS data on the World Wide Web.

**Methods:**

HITO was incrementally developed and implemented. First, classes for the description of software products in health IT evaluation studies were identified. Second, requirements for describing LIFOSS were elicited by interviewing domain experts. Third, to describe domain-specific functionalities of software products, existing catalogues of features and enterprise functions were analyzed and integrated into the HITO knowledge base. As a proof of concept, HITO was used to describe 25 LIFOSS products.

**Results:**

HITO provides a defined set of classes and their relationships to describe LIFOSS in medicine and health care. With the help of linked or integrated catalogues for languages, programming languages, licenses, features, and enterprise functions, the functionalities of LIFOSS can be precisely described and compared. We publish HITO and the LIFOSS descriptions as LOD; they can be queried and viewed using different Semantic Web tools, such as Resource Description Framework (RDF) browsers, SPARQL Protocol and RDF Query Language (SPARQL) queries, and faceted searches. The advantages of providing HITO as LOD are demonstrated by practical examples.

**Conclusions:**

HITO is a building block to achieving unambiguous communication among health IT professionals and researchers. Providing LIFOSS product information as LOD enables barrier-free and easy access to data that are often hidden in user manuals of software products or are not available at all. Efforts to establish a unique terminology of medical and health informatics should be further supported and continued.

## Introduction

### Background

Libre/free and open-source software (LIFOSS) products are increasingly used to support various tasks in health care. LIFOSS generally refers to software products with openly available source code that users and developers can view, analyze, modify, and redistribute.

For example, there are free and open-source software products to implement radiology information systems, picture archiving and communication systems (PACS), patient administration systems, and electronic health record (EHR) systems. Especially in low-resource settings, using LIFOSS can help establish computer-based health information systems [1-3]. Along with their use in hospital and medical practice settings, LIFOSS products are available for mobile health, telemedicine, and public health (eg, [4,5]). Moreover, the COVID-19 pandemic has led to the development of numerous mobile applications for contact tracing, risk assessment, or appointment scheduling, which are often based on LIFOSS and used both in low-resource settings and industrial countries [6,7]. Since 2010, the Medfloss.org database has provided descriptions of LIFOSS used in health care and medicine [8]. As of October 2022, it lists 385 software products and describes them by characteristics like “license,” “application type,” “enterprise function,” “language,” “platform,” and “home page.” Due to the iterative and sometimes uncontrolled growth of the self-developed nomenclature over the last few years, there are several inconsistencies in the software descriptions on Medfloss.org. First, there are misleading assignments of descriptors to characteristics. For example, “laboratory,” “cellular networks,” and “virtual reality” are listed as enterprise functions supported by a software product. However, they describe the setting where the software might be used or special features of the software. Second, the lack of uniform terminology in health informatics has led to the use of synonyms or overlapping terms. For example, the borders between “electronic health systems,” “electronic medical record systems,” and “hospital management systems” are not clearly defined, sometimes leading to ambiguous descriptions of software products. The lack of uniform terminology for describing medical and health care software products and LIFOSS is also apparent when analyzing comparisons of LIFOSS for EHR systems. In several studies published during the last 15 years [1-3,9-11], each research group selected different criteria and descriptors for comparing the technical and functional characteristics of EHR systems.

Indeed, the lack of uniform terminology is not restricted to LIFOSS. There are already several terminologies in health care and health informatics that could potentially be used to describe software. For example, in addition to allowing for precise descriptions of disease patterns, SNOMED (Systematized Nomenclature of Medicine) lists health occupations and environments that may describe the health facilities or settings in which LIFOSS is used. The World Health Organization (WHO) classifies digital health interventions by hierarchies or lists of clients, health care providers, health system managers, data service features, and application system categories [12]. The Health Level 7 (HL7) EHR System Functional Model is a comprehensive catalogue of EHR systems’ features [13]. Finally, some health informatics textbooks provide systematic collections of application systems in health care and their typical features (eg, [14]). However, at first sight, these different terminologies are not easily comparable with each other due to the use of synonyms or homonyms. Linking these different terminologies requires semantic analyses based on a uniform set of concepts, along with easy-to-use methods and tools to link these data.

Linked Open Data (LOD) are regarded as the state-of-the-art principle for linking and structuring concepts from different terminologies. LOD are identifiable by a URI and provided using the Resource Description Framework (RDF) standard [15].

### Benefits of Unified LIFOSS Terminology Using LOD

A unified LIFOSS terminology using LOD has several advantages. First, the use of predefined and open terminologies supports the search for and comparison of software products. Second, further knowledge, such as results of assessment and evaluation studies, can be linked easily to the software descriptions and thus support evidence-based health informatics. For example, linking systematic descriptions of software products with descriptors from other projects can support ontology-based approaches to software requirements engineering [16].

The Austrian-German research project “Health Information Technology Ontology (HITO)” aims to systematically describe software products and their installations in health care. It uses an underlying ontology named HITO, LOD methods and tools, and freely available catalogues to describe software characteristics. HITO is developed based on different use cases in which precise software descriptions are needed, such as when selecting LIFOSS or commercial software products, searching for evidence about the installation of software products, and communicating about software products among stakeholders in health care. In this paper, we focus on LIFOSS products and describe them with the help of openly available catalogues. Especially for LIFOSS products, information about software characteristics is freely available, and LIFOSS developers are likely to recognize the potential advantage of spreading knowledge about their products with the help of LOD.

### Objectives

This study aims to (1) describe LIFOSS systematically with the help of precise descriptors that are captured in HITO and (2) publish HITO and HITO-based software product descriptions as LOD using Semantic Web tools for viewing and querying LIFOSS data on the World Wide Web.

## Methods

### Requirements Elicitation

#### Initial Steps

As the first step toward precise descriptions of software products, the classes and relationships that are useful to describe software products had to be identified. HITO was developed and refined iteratively by collecting requirements of several use cases. Each use case focused on a situation in which a clear terminology for software products was considered essential. These use cases dealt with the description of LIFOSS or commercial software products for potential and current users in health care settings or the description of software product installations in evaluation studies on health IT interventions. We selected diverse use cases to identify the most relevant characteristics for these diverse situations. Based on these use cases, we incrementally built the ontology that contains a general pattern for describing software products in health care.

#### Use Case 1: Evaluation of Digital Health Interventions

The first HITO use case dealt with evaluation studies in health informatics. In evaluation studies, it is crucial to carefully describe the intervention in a reproducible and clear manner to allow generalizability of the findings and their aggregation, for example, in the form of systematic reviews. We used an inductive approach and extracted software descriptions from 24 randomly selected published health IT evaluation studies [17]. We found that software product installations were mainly described by their features, application system type, organizational units where they are used, and user groups. These characteristics were specified as classes and added to HITO (Table 1). Altogether, the software descriptions found in evaluation studies are sparse and concentrate on selected evaluated features. Evaluation studies also often use inconsistent terms, which motivated us to develop a clear terminology for software products.

**Table 1:** HITO^a^ classes to describe software products in health IT evaluation studies (HITO use case 1).

HITO class (characteristic of software products)	Description and examples
Software product	Piece of software that is sold as a commercial product or distributed under an open-source license
Feature	Functionalities offered by a software product that directly contribute to the fulfillment of 1 or more enterprise functions (eg, email notification of new results, user directory to control any access)
Application system type	Commonly used names for categories of software product installations in health care (eg, radiology information system, CPOE^b^ system)
Organizational unit	Health care setting in which the software product is used and an evaluation study was conducted (eg, laboratory, department of pediatrics)
User group	Health care staff who uses the software product installation (eg, nurse, radiologist)

^a^HITO: Health Information Technology Ontology.

^b^CPOE: computerized physician order entry.

#### Use Case 2: Description of LIFOSS With Medfloss.org Project Database

In the LIFOSS use case, we extended the list of HITO classes by carefully analyzing the Medfloss.org project database. Medfloss.org aims to offer an overview of LIFOSS projects related to medical informatics and health care [8]. Although Medfloss.org is not maintained anymore, it is still provided in cooperation with 3 LIFOSS-related working groups of the International Medical Informatics Association, the European Federation for Medical Informatics Association, and the International Society for Telemedicine and eHealth. Within Medfloss.org, the LIFOSS products are described by using a predefined set of categories.

We started this use case by surveying 2 operators of the Medfloss.org database. They were asked independently to answer a survey with 11 open questions and 1 closed question. The survey asked about the users of Medfloss.org, the relevance of the categories used to describe software products, and the positive and negative experiences with the categories used to describe LIFOSS.

The results of this survey show that Medfloss.org is intended to be used by physicians or other health care staff, IT administrators, information managers, and software developers to select appropriate LIFOSS for certain health care tasks. Each LIFOSS product is described by 11 categories, such as “enterprise function,” “application type,” and “license,” on the platform. For each category, the list of descriptors has grown over the years as new LIFOSS descriptions were added.

The answers of the platform operators dealing with the assessment of the current terminology and its representation on the platform were arranged according to a SWOT (Strengths, Weaknesses, Opportunities, and Threats) analysis (Textbox 1).

Both operators rated the “enterprise function,” “application type,” “status,” “license,” “standard,” “language,” “client type,” and “platform” categories of Medfloss.org terminology as “important” or “very important” for the description of LIFOSS. The categories “popularity,” “database,” and “programming language/toolkit” were rated less important by 1 of the website operators.

The strengths of the Medfloss.org terminology used include the rough categorization of software products by application system types and the faceted search on the website that is based on categories for describing the software. However, the survey showed that the categories currently lack the possibility to describe the functionalities and application types with enough precision. Therefore, an opportunity to support Medfloss.org terminology may be to integrate existing terminologies such as WHO’s classification of digital health interventions [12] or the HL7 EHR System Functional Model [13]. Furthermore, the folksonomy (ie, the collection of users’ tags for certain objects) of the platform users is not handled by the search functions on Medfloss.org [18].

Based on the SWOT analysis, we selected 9 (out of 10) of the Medfloss.org categories and added these to HITO (Table 2). Some classes used on Medfloss.org were renamed, such as “standard,” which was changed to “interoperability standard” to sum up interoperability standards, as well as frameworks describing how to use interoperability standards, such as Integrating the Healthcare Enterprise (IHE). A more fine-grained classification according to interoperability levels was examined for common interoperability standards but proved to be impractical. Many interoperability standards such as HL7 Fast Healthcare Interoperability Services (FHIR) can be assigned to multiple interoperability levels. The Medfloss.org category “programming language/toolkit” was split into 2 classes to distinguish between these different concepts.

**SWOT (Strengths, Weaknesses, Opportunities, and Threats) analysis for Medfloss.org.**

**Strengths**

- Current set of categories to describe libre/free and open-source software
- Rough categorization by application system types
- Usefulness of the categories to provide a faceted search on the platform

**Weaknesses**

- Conceptual overlaps in categories (eg, electronic health record and electronic medical record)
- Missing hierarchies for enterprise functions
- Missing detailed functional descriptions

**Opportunities**

- Enhancements of categories by existing terminologies seems possible
- Modeling of user group–dependent categories may increase usefulness

**Threats**

- No handling of synonyms within users’ search terms

**Table 2:** HITO^a^ to describe LIFOSS^b^ products (HITO use case 2).

Medfloss.org class name	HITO class	Description and examples
Client type	Client	The client type on which a software product can be run (mobile, native, or web).
Database	Database management system	Some examples are PostgreSQL^c^ or MySQL.
Enterprise function	Enterprise function	Describes what action humans or machines must carry out in a certain enterprise to contribute to its mission or goals (eg, patient admission, order entry).
Home page	Home page	Home page of the software product or its development project.
Standard	Interoperability standard	Ability of 2 or more components to exchange information and to use the information that has been exchanged. Under this class name, interoperability standards (eg, HL7^d^ FHIR^e^ or DICOM^f^) or frameworks describing how to use standards (eg, IHE^g^) are summed up.
Language	Language	Languages in which the software product is available (eg, English, French, and German).
License	License	The license under which a software product is distributed.
Platform	Operating system	The operating system a software uses (eg, Windows). A software product might be able to run on a variety of operating systems.
Programming language/toolkit	Programming language	The programming language used to develop a software product (eg, Java or Python).
Programming language/toolkit	Programming library or toolkit	Programming toolkits are utility programs that are used to develop and maintain software. Programming libraries are a collection of prewritten functions that are ready to be used in coding. Both help programmers develop software in a fast and safe manner.

^a^HITO: Health Information Technology Ontology.

^b^LIFOSS: libre/free and open-source software.

^c^SQL: Structured Query Language.

^d^HL7: Health Level 7.

^e^FHIR: Fast Healthcare Interoperability Resources.

^f^DICOM: Digital Imaging and Communications in Medicine.

^g^IHE: Integrating the Healthcare Enterprise.

#### Further HITO Use Cases

We summarized 3 further use cases that have not elicited new HITO classes related to software product characteristics.

The third use case deals with the description of commercial software products used in health care. This use case confirmed the set of HITO classes that we already identified by describing LIFOSS products. However, for commercial software products, it is quite challenging to describe them based on these classes because meaningful descriptions of commercial software products are rarely publicly available.

In the fourth use case, the existing HITO classes were linked with competency levels for IT staff in health care organizations, which might be useful for creating job advertisements.

In the fifth use case, findings from the HITO project were discussed with practitioners like hospital chief information officers and industry representatives to discuss the applicability and broader use of the HITO project’s findings in practice.

### Software Product Descriptions as LOD

After building HITO from the described use cases, we published HITO and HITO-based software product descriptions using LOD.

LOD are web data with an open license. They allow the use of Semantic Web tools for viewing and querying LIFOSS data on the World Wide Web. LOD also allow for linking and reusing existing terminologies. To be considered “5-star LOD,” the data need to be machine-readable, presented in a nonproprietary format, use open standards of the World Wide Web Consortium (W3C), and be linked to other data [15]. We strived to achieve 5-star linked open HITO data.

Accordingly, we used RDF Schema (RDFS) and the Web Ontology Language (OWL). RDFS and OWL are W3C standards used to define the types of elements of discourse as classes. The *class* “software product,” for example, represents the set of all individual software products. *Properties* represent possible binary-typed relationships between individuals of certain classes, for example, between software products and features. Using RDF, facts (relationships between individuals) are expressed as subject-predicate-object triples, whose elements may be defined and stored in different places. This allows for reusing existing vocabularies and interlinking with existing knowledge bases, forming the LOD cloud. Each RDF resource (a class, an individual, or a relationship) has a URL where it is published both in human-readable (ie, HTML) format and in machine-processable RDF serialization format. To browse RDF data comfortably, tools like RickView [19] can be used and modified. SPARQL Protocol and RDF Query Language (SPARQL) end points allow free access to structured read-only queries for humans and as application programming interfaces (APIs) for several tools.

### Integration of Software Product and Health-Related Terminologies

One advantage of LOD is their easy integration of existing data sources that are already available in RDF format. Therefore, for the HITO classes (Tables 1 and 2) that characterize software products, we searched for “catalogues” (ie, lists of terms that can be used as instances for the respective class). For the selection of suitable catalogues, we defined the following criteria:

[The catalogue should be authored by an established scientific or standardization organization.] OR [The catalogue must be scientifically plausible with regard to reproducibility, having undergone peer-review or having been developed by more than 5 persons.] OR [The catalogue must be openly available and developed by a large community.]

We used a broad literature and web search and our knowledge of the field to identify related taxonomies and catalogues that can be useful for describing instances of HITO classes (Table 2). In the following paragraphs, we provide a brief overview of the catalogues we investigated.

We started with DBpedia, a popular and large knowledge base comprising billions of triples extracted from Wikipedia, texts, and other sources [20,21]. We analyzed DBpedia to identify possible instances or subclasses for the HITO classes. For the classes “language,” “operating system,” and “programming language,” we found DBpedia classes with suitable instances that we replaced the associated HITO classes with to increase interoperability. Other instances of DBpedia classes, such as the “license” class, were not suitable for integration because DBpedia does not semantically differentiate between licenses for software products and licenses for other purposes, like drivers’ licenses. For software product licenses, we integrated subclasses of the class “open-source software license” derived from the Software Ontology for biomedical software [22].

The most challenging task was the integration of catalogues for the classes “application system type,” “enterprise function,” “feature,” “organizational unit,” and “user group,” for which we needed instances or subclasses related to health care. We checked the following sources for the integration of catalogues into HITO:

- HL7 EHR System Functional Model [13]: Using the examples of 2 installations of commercial software products for EHR systems, we assessed whether the features of the software product could be described by this model. We found that the whole list of conformance criteria defined in this model would be too detailed for a HITO catalogue. However, the section labels that are provided by this model, such as “manage allergy, intolerance, and adverse reaction list,” provide an appropriate level of detail for feature descriptions in HITO.
- The textbook by Winter et al [14] describes a set of application system types and a set of enterprise functions in hospitals. An analysis of Medfloss.org revealed that the sets of enterprise functions or application system types could be used to tag 71% and 42%, respectively, of 356 Medfloss.org software products analyzed in use case 2 [23]. Due to the textbook’s focus on hospitals, appropriate terms for software products used in other health institutions or for public health were missing.
- Taxonomy for health IT by Varshney et al [24]: The application system types identified by Varshney and colleagues proved to be too coarse-grained for classification. Only 105 (29%) of 356 Medfloss.org software product entries could be classified by the taxonomy [23].
- WHO classification of digital health interventions [12]: This multiaxial classification lists system categories and interventions for clients, health care providers, health system managers, and data services. The 25 system categories correspond to application system types in HITO, and their strength lies in their focus on application systems for health care networks rather than for single institutions. For the hierarchically grouped interventions such as “2.10 laboratory and diagnostics imaging management” and “2.10.1 transmit diagnostic result to healthcare provider,” a more differentiated assignment to functions and features in the context of HITO is needed. Therefore, “2.10 laboratory and diagnostics imaging management” is listed as an enterprise function in HITO, whereas “2.10.1 transmit diagnostic result to healthcare provider” is listed as a feature in HITO.
- SNOMED Clinical Terms (CT) [25]: For the 24 evaluation studies analyzed in use case 1, we found that the software products’ user groups or the organizational units where the software products are used can sufficiently be described by subclasses of SNOMED CT’s “occupation” or “environment” classes, respectively.
- Features for PACS selection [26]: The PACS features described on 3 hierarchy levels were transformed into a flat list of 38 features useful for describing examples of PACS software products.
- The LIS (laboratory information system) Functionality Assessment Toolkit of the Association for Pathology Informatics [27]: This toolkit lists 850 functionality statements and describes a methodology for selecting a LIS. In comparison to the other feature catalogues we described, the functionality statements are very detailed. For integration into HITO, a clustering of the functionality statements would be necessary to obtain a manageable LIS feature list.

### Describing Software Products Using HITO

With the help of HITO and the selected catalogues we described, 25 LIFOSS products were described. These were selected to represent different application system types and due to their comprehensive, openly available documentation. For the description of supported enterprise functions and features, we extracted terms from the software product documentation and linked as many as possible to catalogue entries of HITO. Information about the LIFOSS products was extracted by 1 team member (author MB) and checked by another team member (author FJ).

## Results

**Figure 1:**
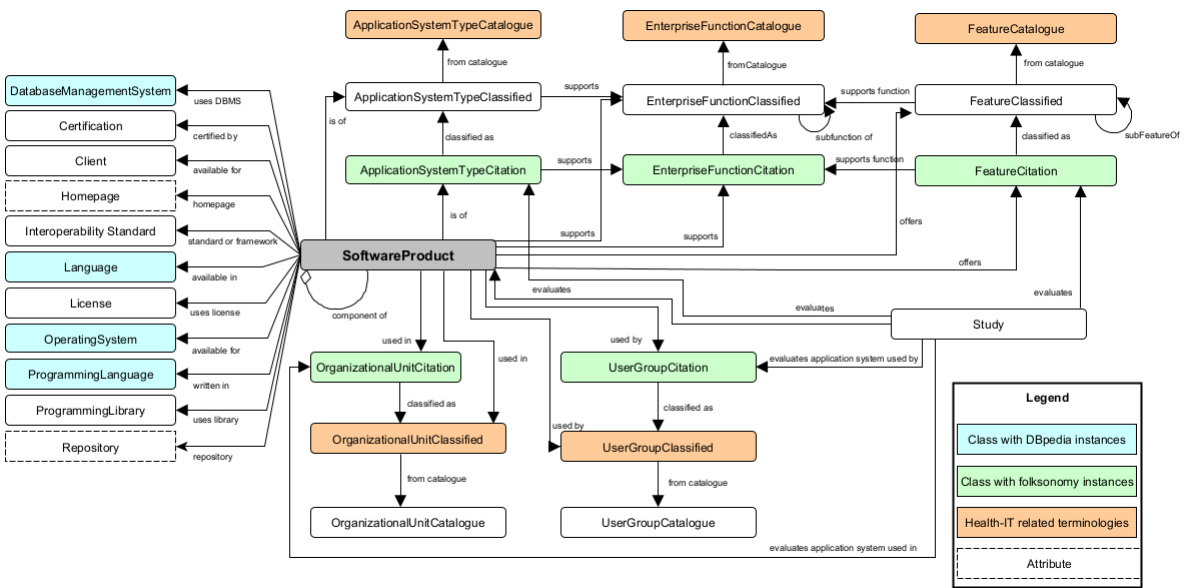
The Health Information Technology Ontology (HITO), version 22.05, specifies the classes and relationships that are used to describe software products. The complete class diagram is available on the HITO website [28].

HITO was modeled and published using Semantic Web technologies. The Unified Modeling Language class diagram in Figure 1 describes the structure of HITO. The classes shown to the left of “software product” describe the general characteristics of software products. For classes with domain-specific instances (ie, application system type, feature, enterprise function, organizational unit, and user group), we applied a scheme of 3 interrelated classes named *<classname>*Catalogue, *<classname>*Classified, and *<classname>*Citation. A *<classname>*Catalogue is a health IT–related collection, such as the HL7 EHR Functional Model or the WHO classifications for digital interventions. A *<classname>*Classified is a category that belongs to exactly 1 catalogue. A *<classname>*Citation is a textual label extracted from available software manuals, descriptions, and studies and thus represents a part of the folksonomy contained in HITO [28].

To illustrate how this scheme applies, we describe the domain-specific characteristics of the Orthanc software [29,30] in Table 3. The developers of Orthanc refer to it as “mini-PACS,” “DICOM (Digital Imaging and Communications in Medicine) server,” “VNA (vendor-neutral archive),” and “viewer of medical images.” These terms are assigned to the software product Orthanc as “application system type citations.” In turn, “DICOM server” and “mini-PACS” have links to the classified application system type “PACS” from the application system type catalogue in Winter et al [14]. The supported enterprise function citations extracted from the Orthanc website, such as “image archiving,” “image management,” and “research about the automated analysis of medical images,” were linked to the more general terms “laboratory and diagnostics imaging management” and “research and education” from enterprise function catalogues. For the 16 feature citations extracted from the Orthanc online documentation, we identified linkable classified features of the WHO classification of digital health interventions and the PACS feature list. Some classified features or enterprise function terms have direct links to the software product. These assignments had no match with citations and were done by domain experts.

**Figure 2:**
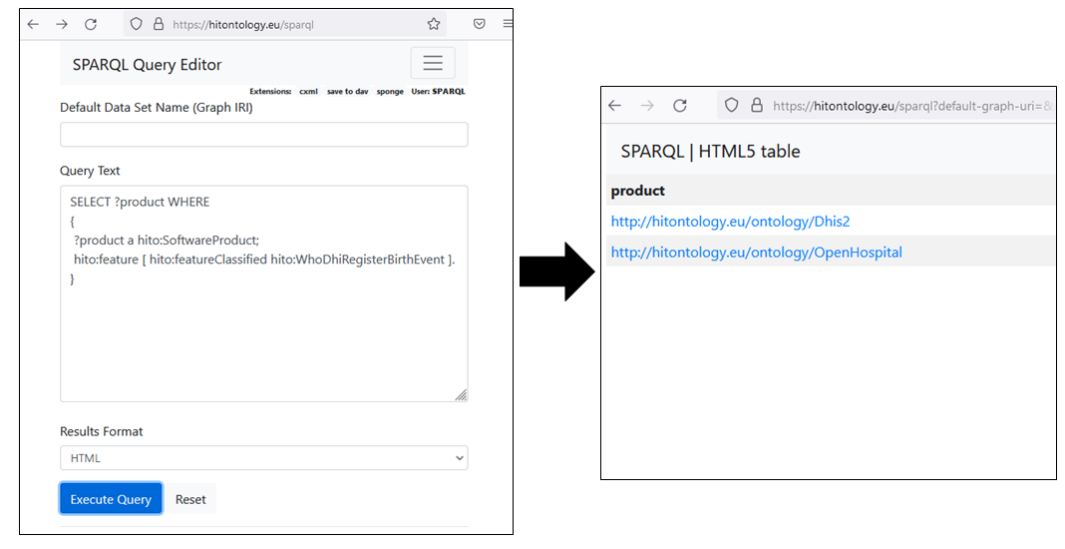
A SPARQL Protocol and RDF Query Language (SPARQL) query (on the left) and its results (on the right). RDF: Resource Description Framework.

Overall, with the help of HITO, we described 25 LIFOSS products in similar detail as Orthanc. All software product descriptions are available as LOD. We described HITO classes and relationships of the ontology and individual software products using RDF. The HITO SPARQL end point [31] allows queries using SPARQL (Figure 2).

**Figure 3:**
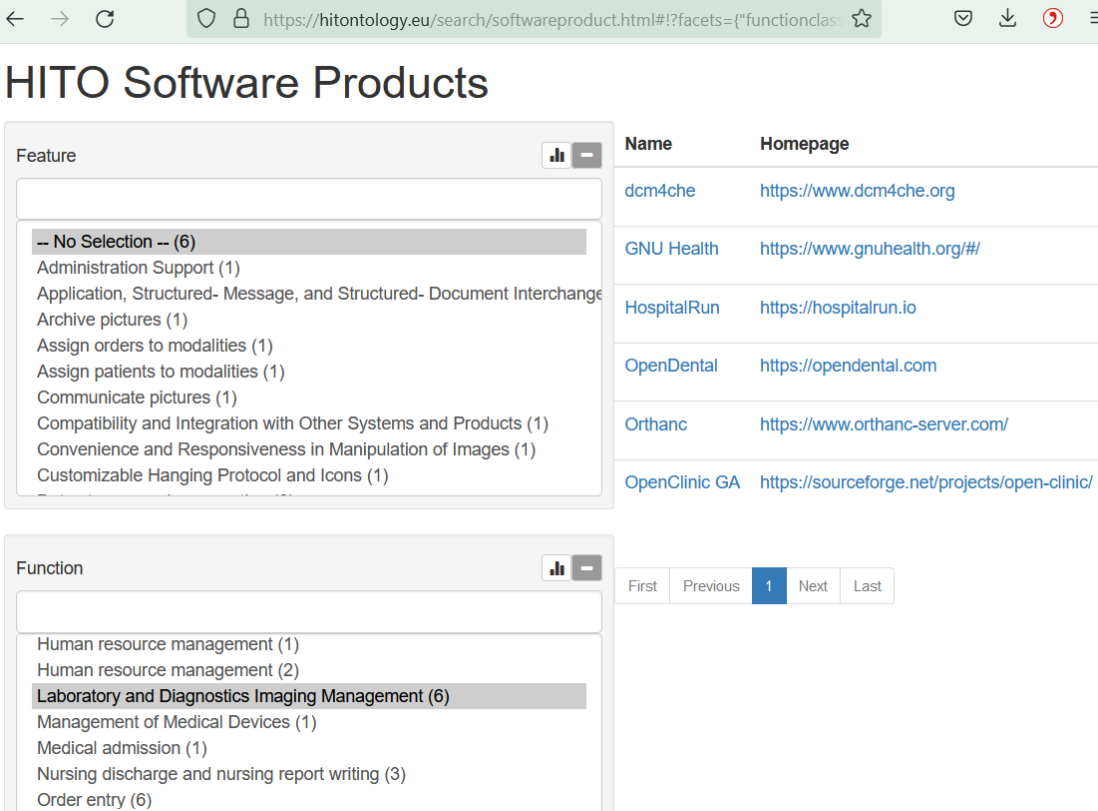
Faceted search for software products supporting “Laboratory and Diagnostics Imaging Management”. HITO: Health Information Technology Ontology.

The RickView application allows for browsing through the ontology and knowledge base [32]. Another suitable way to query is with a faceted search [33], whereby integrated terminologies can be used to find software products of a certain application system type or supporting combinations of features and functions (Figure 3).

The ontology is also publicly available under version control in a GitHub repository [34]. It can be downloaded in RDF Turtle format to be viewed in ontology editors like Protégé. HITO is dedicated to the public domain and uses a Creative Commons Zero v1.0 Universal license. There are a few exceptions for integrated terms from SNOMED [25] and the WHO classification of digital health interventions [12].

**Table 3:** Application system types, supported functions, features, user groups and organizational units of the Orthanc software.

<className> and <classname>Citation from the software documentation		<classname>Classified in HITO^a^ (<classname>Catalogue)
**Application system type**		
	DICOM^b^ server	PACS^c^ (application systems in [14])
	mini-PACS	PACS (application systems in [14])
	VNA^d^	N/A^e^
	Viewer of medical images	N/A
	Web viewer	N/A
**Enterprise function**		
	N/A	Execution of radiological examinations (enterprise functions from [14])
	Image archiving	Laboratory and diagnostics imaging management (enterprise functions from [12])
	Image communication	Laboratory and diagnostics imaging management (enterprise functions from [12])
	Image distribution	Laboratory and diagnostics imaging management (enterprise functions from [12])
	Image management	Laboratory and diagnostics imaging management (enterprise functions from [12])
	Research about the automated analysis of medical images	Research and education (enterprise functions from [14])
**Feature**		
	N/A	Capture diagnostic results from digital devices (features from [12])
	Data management for clinical routine and medical research	N/A
	DICOM identifiers	N/A
	DICOM network protocol	N/A
	Evaluations Report	N/A
	Injury surveillance system registration report	N/A
	Listing available servers	N/A
	Plugin mechanism to add new modules	N/A
	Retrieve images	N/A
	Retrieving DICOM resources from WADO-RS^f^ server	Compatibility and integration with other systems and products (PACS feature list [26])
	Search the content	N/A
	Send images	N/A
	Sending DICOM resources to a STOW-RS^g^ server	Compatibility and integration with other systems and products (PACS feature list [26])
	Test the connection	N/A
	Top diseases report	N/A
**User group**		
	Radiologist	Radiologist occupation [25]
	Researcher	Researcher occupation [25]
	Software/hardware integrators in the medical field	N/A
	Network engineer	N/A
	Physicist	Physicist occupation [25]
	System engineer	N/A
**Organizational unit**		
	Health centers	Health center environment [25]
	Hospital environment	Hospital environment [25]
	Radiology department	Radiology department environment [25]

^a^HITO: Health Information Technology Ontology.

^b^DICOM: Digital Imaging and Communications in Medicine.

^c^PACS: picture archiving and communication system.

^d^VNA: vendor-neutral archive.

^e^N/A: not applicable.

^f^WADO-RS: Web Access to DICOM Objects by RESTful (representational state transfer) Services.

^g^STOW-RS: Store Over the Web by RESTful Services.

## Discussion

### Principal Results

In this project, we identified characteristics useful for describing software products and systematically captured them as classes in HITO. Accordingly, we exploited the properties of ontologies that enable semantic description and linking of data.

For a thorough functional description of software products in health care, we described the enterprise functions supported and features offered by the software products. For health-specific characteristics of software products, we analyzed and integrated existing terminologies for enterprise functions, features, application system types, organizational units, and user groups as catalogues. Because we expected the relevant sources for such catalogues to come not only from science but also from practice, a systematic review of the scientific literature for health IT–related terminologies would not have led to sufficient results. Accordingly, we based the selection of catalogues on statements by domain experts among the project team and project partners, supplemented by targeted PubMed and Google searches on specific application system types.

Thus, we used a case-based and agile approach to identify classes, relationships, and catalogues best suited for describing software products in health care. In the use cases considered so far, ontological reasoning had no relevance. Therefore, to date, only few axioms are used in HITO. As the next step toward more interoperability with other formal ontologies, HITO could use an upper-level ontology such as Basic Formal Ontology [35], General Formal Ontology [36], or gist [37]. A first feasibility check of these ontologies showed that the gist ontology, which defines typical upper-level business concepts, may be the most appropriate for the scope of HITO.

With the help of HITO, we described 25 LIFOSS products in detail that, together with less detailed descriptions of single commercial software products and software products extracted from evaluation studies, form our knowledge base. The descriptions of these software products could be regarded as a proof of concept. However, we noticed the interpretative degrees of freedom in assigning correct enterprise functions and features to software products. To ensure the validity of further software descriptions, it would be helpful to calculate interrater reliability among 2 independent experts. For this, further software product entries of the Medfloss.org database that have not yet been considered in HITO’s knowledge base could be used.

As postulated by Berners-Lee [15], HITO’s availability as LOD facilitates its barrier-free access and use. In particular, the integrated catalogues for enterprise functions, features, and application system types provide HITO users with rich terminology for functionalities of software products. However, since there is more than 1 catalogue for each of these characteristics, new terminological problems arise. The catalogue entries of different catalogues must be mapped to each other to achieve comparability of software products described with the help of different catalogues. Linking these catalogues is part of ongoing research. Together with the folksonomy terms that are already connected to catalogue entries, HITO users will be able to retrieve the most suitable software using a broad range of search terms. The catalogues currently integrated into HITO focus more on health care rather than on medical research tasks (ie, software products like research databases may not be sufficiently described by HITO). However, integrating further catalogues describing research-related enterprise functions or features could be possible.

Nevertheless, publishing HITO and its knowledge base as LOD implies that the contents of HITO are available under an open license. Thus, for the broadly accepted nomenclature SNOMED CT, we could only check the principal suitability of the SNOMED “environment” and “occupation” concepts based on a small set of examples that we included in HITO with permission from SNOMED International. Due to license requirements, SNOMED CT terms cannot be made available as LOD.

In summary, HITO provides an openly available framework for the description of health care–related software that can be used by researchers who publish studies on digital health interventions or by developers and users who need to describe software.

### Conclusions

We recognize that health informatics continues to face a terminology problem. Establishing a uniform terminology for software products used in health care is currently unachievable due to several coexisting terminologies from both research and practice. Linking the terms from different terminologies by similarity relationships is the first step toward more transparency. This will also help identify misunderstandings that may be caused by synonyms, homonyms, or conceptual overlaps. Simply knowing that the term “EHR system” can stand for an institutional or cross-institutional application system or a collection of digital documents related to a person's health prevents problems related to misunderstanding. A researcher authoring a study on a digital health intervention by an EHR system knows that the term “EHR system” must be further specified, for example, by enterprise functions and features as listed in HITO.

Nevertheless, we should further strive for a consented, uniform terminology of health informatics. Taking different coexisting terminologies as a basis, methods of qualitative content analyses such as inductive category formation [38], supported by (semi)automatic text extraction, may lead the way toward an established language for health informatics.

## Abbreviations

API: application programming interface
DICOM: Digital Imaging and Communications in Medicine
EHR: electronic health record
HITO: Health Information Technology Ontology
HL7: Health Level 7
LIFOSS: libre/free and open-source software
LIS: laboratory information system
LOD: Linked Open Data
OWL: Web Ontology Language
PACS: picture archiving and communication system
RDF: Resource Description Framework
RDFS: Resource Description Framework Schema
SNOMED: Systematized Nomenclature of Medicine
SNOMED CT: Systematized Nomenclature of Medicine Clinical Terms
SPARQL: SPARQL Protocol and RDF Query Language
SWOT: Strengths, Weaknesses, Opportunities, and Threats
VNA: vendor-neutral archive
W3C: World Wide Web Consortium
WHO: World Health Organization
